# 40008 COMBINED CRISPR/CAS9 AND AAV FOR THE GENERATION OF CONDITIONAL ISOGENIC GENE KNOCK-INS

**DOI:** 10.1017/cts.2021.460

**Published:** 2021-03-30

**Authors:** Prajwal Boddu, Abhishek Gupta, Todd Waldman, Manoj Pillai

## Abstract

ABSTRACT IMPACT: We describe a novel methodology that combines CRISPR/Cas9-induced double stranded DNA breaks with homology dependent repair from an adeno associated virus (AAV) encoded template to generate single-allele edited isogenic cell line models of cancer-associated mutations with high efficiency. OBJECTIVES/GOALS: Conventional approaches to creating isogenic knock-ins, to model disease-associated mutations, are limited by poor efficiency and loss of the mutant allele on extended culture. We present an optimized editing approach combining CRISPR/Cas9 with Adeno-associated virus, using mutant SF3B1 as a prototype. METHODS/STUDY POPULATION: Left and right homology arms for SF3B1 were PCR amplified and cloned into pAAV-SEPT-Acceptor plasmid (containing a chimeric intron, neomycin resistance cassette and polyA tail). The disease-associated K700E mutation was introduced by site-directed mutagenesis. Single guide RNA (sgRNA) complexed with recombinant Cas9 along with the AAV donor were delivered into K562 cells, G418 resistant clones selected, and screened for integration by PCR. Confirmed clones were then transduced with a doxycycline-inducible Cre-recombinase containing lentiviral vector. Inducible expression of Cre-recombinase and expression of the mutant allele were confirmed by Western blot and Sanger sequencing respectively. RESULTS/ANTICIPATED RESULTS: Targeted-integration efficiencies among the Neo-resistant clones, generated by AAV-alone and AAV+CRISPR/Cas9, were 16% and 94%, respectively. Single cell cloning after Cre-mediated excision of loxp was unsuccessful presumably due to toxicity of the K700E mutation. To overcome this limitation, clones were transduced with doxycycline-inducible Cre-recombinase lentiviral vector. Doxycycline induction of Cre-recombinase resulted in reliable excision of the loxp cassette and expression of mutant allele at about 50% variable allele frequency (as determined by Sanger sequencing). The approach was validated in additional cell lines and for introduction of N-terminal FLAG tag for SF3B1. DISCUSSION/SIGNIFICANCE OF FINDINGS: Combining AAV and CRISPR/Cas9 can generate scalable single-dominant allele mutants with high precision and efficiency compared to AAV or CRISPR alone. Together with inducible Cre-recombinase, our approach can generate isogenic models where the mutation confers a growth disadvantage.

